# The differences in intestinal flora and metabolites between H-type hypertension and non-H-type hypertension

**DOI:** 10.1186/s12967-025-06295-8

**Published:** 2025-03-14

**Authors:** Jian Wu, Jiangman Zhao, Yinhong Cheng, Haoliang Zhou, Guanqiao Shen, Hongying Ding, Jin Lv, Shiye Dong, Oushan Tang

**Affiliations:** 1 Department of Cardiovascular Medicine, Shaoxing Second hospital, Shaoxing, Zhejiang China; 2 Shanghai Biotecan Pharmaceuticals Co., Ltd, Shanghai, China; 3 Shanghai Zhangjiang Institute of Medical Innovation, Shanghai, China

**Keywords:** H-type hypertension, Gut microbiota, Short-chain fatty acids, Trimethylamine N-oxide, N-acetylglutamine

## Abstract

**Objective:**

In order to explore the differences in gut microbiota and their metabolites between patients with H-type hypertension and non-H-type hypertension.

**Method:**

Our study included 100 hypertensive patients from the Department of Cardiology at Shaoxing Second Hospital, with 51 patients having H-type hypertension (H group) and 49 having non-H-type hypertension (non-H group). Blood samples were collected for clinical and metabolite testing, and fecal samples were collected for 16 S rRNA sequencing and metabolite testing.

**Results:**

No significant difference in the α and β diversity of the gut microbiota between the two groups of patients were observed. However, at the phylum level, patients in the non-H group have a higher abundance of Firmicutes and a lower abundance of Actinobacteriota. At the genus level, compared to the non-H group, the H-type group has a higher abundance of Bifidobacterium; at the species level, the Non-H group has a higher abundance of Bacteroides vulgatus and lower abundances of Bacteroides stercoris and Bacteroides plebeius. In the serum of both groups, the concentrations of Acetate and Isobutyrate were significantly higher in the H group (*P* < 0.05), while in the feces of both groups of patients, the concentration of Malonate was significantly higher in the Non-H group.

**Conclusion:**

The microbial sequencing shows distinct differences between the H and non-H groups, with the latter having higher Firmicutes and Bacteroides vulgatus, while the H group has more Bifidobacterium and higher serum acetate levels. These variations suggest unique gut microbiota compositions and metabolite profiles for each group.

## Introduction

Hypertension is a major contributor to the increasing global disease burden, with over 1.3 billion people worldwide suffering from it, accounting for about 31% of the adult population globally. It is the primary risk factor for the onset of cardiovascular and cerebrovascular diseases and is an important cause of disability and death worldwide [1]. In China, around 75% of hypertensive patients also have elevated homocysteine (Hcy) levels (≥ 10 µmol/L), forming a subgroup known as H-hypertension. Elevated Hcy directly impacts blood pressure, with higher concentrations correlating to increased blood pressure. The combination of high Hcy and hypertension amplifies arteriosclerosis and stroke [2, 3]. Studies show that the risk of stroke induced by H-type hypertension is four times higher than that in simple hypertension. Ischemic stroke (IS), which constitutes about 80% of all stroke cases, is strongly associated with high Hcy levels. Elevated Hcy contributes to IS by damaging endothelial function, releasing neurotoxins, and promoting thrombus formation [4, 5].

The mechanisms underlying hypertension are complex, and research has found that it may be related to genetics, environment, as well as gut microbiota [6–11]. Studies have shown that patients with hypertension exhibit significantly reduced gut microbial abundance and diversity, with a notable decline in beneficial bacteria and an overgrowth of Proteus and Klebsiella [12, 13]. Dysbiosis of gut microbiota can cause damage to the intestinal epithelial barrier, trigger systemic inflammation, disrupt gut mechanical force transmission, and influence blood pressure regulation through vascular morphology and function, as well as autonomic nervous system activity [14]. Gut microbial metabolites play a crucial role in the microbiota-host crosstalk, affecting the gut-brain axis and influencing host blood pressure, both independently and via the host immune system. Beneficial metabolites, such as short-chain fatty acids and indole-3-lactic acid, help regulate blood pressure, while trimethylamine N-oxide (TMAO) has a harmful effect. In both healthy individuals and patients with cardiovascular diseases, an increased concentration of TMAO in the circulatory system is observed to have a significant dose-response relationship with an increased risk of hypertension [15]. Using hypertensive animal models, it was found that microbial metabolites such as acetate and propionate, derived from the metabolism of dietary fiber, are associated with lower blood pressure [13]. Additionally, patients at high cardiovascular risk often have a gut microbiota with greater capacity to metabolize methionine, producing homocysteine (Hcy), which can damage vascular endothelial cells and trigger inflammation. However, no research has yet compared gut microbiota and their metabolites between patients with H-type hypertension and non-H-type hypertension. This study aims to investigate the differences in the gut microbiota and their metabolites between these two types of hypertensive patients to provide a new strategy for the treatment of hypertension.

## Materials and methods

### Study population and samples

Our study included 100 patients with hypertension from the Department of Cardiology at the Shaoxing Second hospital, comprising 55 males and 45 females. The patients were further divided into two groups: the H-type hypertension group (H group *n* = 51) and the non-H-type hypertension group (non-H group *n* = 49). According to the Chinese Hypertension Prevention and Treatment Guidelines [16], the diagnostic criteria for hypertension are three separate measurements of systolic blood pressure greater than 140 mmHg and/or diastolic blood pressure greater than 90 mmHg while at rest in the non-same day. Each blood pressure measurement should be repeated at intervals of 30 to 60 s, and the average of the two readings should be taken, with H-type hypertension defined as having Hcy levels ≥ 10 umol/L. All participants provided written informed consent before joining the study, which was approved by the Medical Ethics Committee of the Shaoxing Second hospital.

### Clinical characteristic measurement

The age, gender, body Mass Index (BMI), systolic blood pressure (SBP), diastolic blood pressure (DBP), and heart rate of each participant were recorded. All clinical trials were conducted in the Clinical Pathology Department of the Second Hospital of Shaoxing. Fasting venous blood samples from these patients were collected and preserved for further testing, including clinical markers such as Hcy, folic acid (Fa), fasting blood glucose (FBG), total cholesterol (TC), triglycerides (TG), low-density lipoprotein (LDL), low-density lipoprotein cholesterol (LDLC), and high-density lipoprotein cholesterol (HDLC). Fecal samples were collected in sterile cryogenic tubes and stored at -80 °C for further sequencing analysis.

### DNA extraction and 16 S rRNA gene sequencing

The extraction of microbial genomic DNA from fecal samples was performed using the PowerMax Extraction Kit according to the protocol provided by MoBio Laboratories in Carlsbad, California. Subsequent to extraction, the DNA quantity and purity were assessed through agarose gel electrophoresis and spectrophotometric analysis with a NanoDrop ND-1000 device from Thermo Fisher Scientific in Waltham, Massachusetts. For the amplification of the 16 S V3 and V4 regions, a set of universal primers, specifically the 341 Forward Primer and the 805 Reverse Primer, were employed. The PCR amplification took place in a 50 µL mixture, with an initial denaturation at 98 °C for 30 s, 25 cycles of denaturation at 98 °C for 15 s, annealing at 58 °C for 15 s, extension at 72 °C for 15 s, and a final elongation at 72 °C for one minute. The PCR products were purified using AMPure Xp Beads from Beckman Coulter, based in Indianapolis, Indiana. The DNA concentration was assessed utilizing the PicoGreen dsDNA Assay Kit from Invitrogen, located in Carlsbad, California. Post-concentration determination, the DNA libraries were prepared for sequencing on an Illumina NovaSeq 6000 instrument with a paired-end 2 × 250 bp configuration, after a quantitative analysis conducted by Shanghai Biotecan Pharmaceuticals Co., Ltd in Shanghai, China.

### Data processing, analysis and visualization

The Qiime2 software, version 2023.2.0, was utilized to process the raw sequencing data using the DADA2 pipeline. The initial step involved quality filtering to eliminate adapter and barcode sequences, along with trimming to discard sequences falling below an average quality score of 25. Following this, the sequences were dereplicated, assessed for sequence variants, merged, and checked for chimeras as per the established DADA2 protocol. Any amplicon sequence variant (ASV) detected at a frequency lower than 50 across all samples or present in less than three samples was excluded. The filtered representative sequences and biom-formatted tables were then annotated using the Silva 138.1 database. The completed table and taxonomy data were exported in the form of a biom table and a textual document, respectively. These exports facilitated further analysis, which included the integration of taxa data into the biom-formatted ASV table.

The “microeco” package (version.1.12.0) facilitated the calculation and graphical representation of both alpha-diversity and beta-diversity indices. Additional visualizations like the taxonomic composition bar plot, feature abundance box plot, Venn diagram, and heatmap were also produced using the package. Given that the microbiota’s relative abundance was of interest, the Linear Discriminant Analysis (LDA) effect size (LEfSe) method was utilized to discern differences in the microbiota’s composition. With the alpha value set at 0.01 and the LDA score cut-off at 4, the LEfSe bar plot and related cladogram were crafted using the “microeco” package.

### Statistical analysis

In this study, continuous variables between two groups were analyzed using the Wilcoxon-test. All categorical and ordinal variables were evaluated using the Chi-square test, with the results expressed in frequencies and percentages. The statistical tool used for analysis was SPSS version 26.0.

## Results

### The baseline information and taxonomy characteristics of the two groups

This study included 100 hypertension patients from November 2021 to November 2023, with 51 cases of H-type hypertension and 49 cases of non-H-type hypertension. The basic information of the patients is shown in Table 1. Other laboratory test indicators for the patients are presented in Table 2.

**Table 1:** Patient’s basic information

Feature	H group	Non-H group	*P*
Gender			
Male	36 (70.59%)	19 (38.78%)	0.003**
Female	15 (29.41%)	30 (61.22%)	
Age	71.65 ± 12.46	68.92 ± 10.29	0.11
BMI	23.51 ± 2.71	24.62 ± 2.71	0.049*
Medication history			
Hypoglycemic agents	9 (17.65%)	11 (22.45%)	0.73
Lipid-lowering agents	43 (84.31%)	44 (89.80%)	0.60
Antihypertensive agents	50 (98.04%)	48 (97.96%)	1.00
Family History of Cardiovascular Disease			
Yes	1 (1.96%)	4 (8.16%)	0.34
No	50 (98.04%)	45 (91.84%)	
History of Stroke			
Yes	2 (3.92%)	3 (6.12%)	0.96
No	49 (96.08%)	46 (93.88%)	
History of Diabetes			
Yes	7 (13.73%)	10 (20.41%)	0.53
No	44 (86.27%)	39 (79.59%)	
History of Cardiovascular Surgery			
Yes	15 (29.41%)	5 (10.20%)	0.03*
No	36 (70.59%)	44 (89.80%)	
Smoking History			
Yes	19 (37.35%)	4 (8.16%)	0.0013**
No	32 (62.75%)	45 (91.84%)	
Drinking History			
Yes	15 (29.41%)	4 (8.16%)	0.014*
No	36 (70.59%)	45 (91.84%)	
SBP	144.69 ± 25.46	143.02 ± 21.80	0.76
DBP	80.84 ± 14.68	82.51 ± 11.26	0.53
Hcy	31.90 ± 20.80	11.00 ± 2.45	<0.001***
Fa	5.95 ± 3.76	9.55 ± 4.16	<0.001***

**Table 2:** Patient’s laboratory test results

Feature	H group	Non-H group	*P*
TC	3.85 ± 1.04	4.76 ± 1.14	<0.001***
TG	1.31 ± 0.71	1.55 ± 1.17	0.35
HDLC	1.08 ± 0.28	1.21 ± 0.28	0.02
LDLC	2.53 ± 0.83	3.11 ± 0.88	0.001**
Apolipoprotein AI	1.07 ± 0.22	1.23 ± 0.24	<0.001***
Apolipoprotein B	0.87 ± 0.29	0.97 ± 0.27	0.09
FBG	5.18 ± 1.80	5.62 ± 1.22	0.003
LDL1	23.69 ± 10.50	32.53 ± 13.52	<0.001***
LDL2	18.63 ± 5.93	25.67 ± 10.19	<0.001***
LDL3	8.84 ± 5.59	10.35 ± 6.59	0.22
LDL4	4.29 ± 5.72	3.51 ± 3.83	0.93
LDL5	1.71 ± 4.12	1.06 ± 2.60	0.64
C-reactive protein	10.79 ± 19.90	2.99 ± 6.38	0.002**
Lactate dehydrogenase	192.75 ± 55.41	179.31 ± 71.98	0.04*
Creatine Kinase	123.58 ± 148.01	98.53 ± 187.90	0.04*
Creatine Kinase-MB	13.10 ± 10.61	14.20 ± 16.62	0.32
Alanine Aminotransferase	21.65 ± 18.34	21.37 ± 12.38	0.39
Alkaline Phosphatase	82.98 ± 29.03	74.67 ± 20.77	0.072
Aspartate Aminotransferase	29.80 ± 23.21	27.31 ± 26.23	0.77
Gamma-Glutamyl Transpeptidase	51.53 ± 47.48	35.53 ± 28.57	0.04*
Cholinesterase	6318.55 ± 1922.83	7258.49 ± 1243.07	0.005**
Total Protein	65.28 ± 6.04	65.38 ± 5.93	0.99
Albumin	37.56 ± 3.87	38.92 ± 2.88	0.052
Globulin	27.45 ± 5.04	27.13 ± 3.75	0.6747
Total Bilirubin	18.34 ± 9.17	13.56 ± 4.83	0.02*
Direct Bilirubin	3.91 ± 2.23	2.35 ± 0.93	<0.001***
Indirect Bilirubin	14.43 ± 7.25	11.24 ± 3.99	0.08
Total Bile Acids	7.61 ± 7.18	5.83 ± 4.16	0.30
Amylase	67.61 ± 34.15	62.22 ± 28.13	0.57
Alpha-L-Fucosidase	26.35 ± 8.10	26.10 ± 4.98	0.85
Adenosine Deaminase	9.80 ± 3.82	8.93 ± 2.84	0.30
Retinol Binding Protein	38.80 ± 16.79	38.57 ± 12.87	0.43
Glucosamine	2.01 ± 0.25	2.01 ± 0.20	0.73
Glucose	5.22 ± 1.83	5.58 ± 1.23	0.009**
Urea	11.56 ± 33.46	5.31 ± 1.78	0.01*
Creatinine	94.25 ± 38.93	68.06 ± 16.76	<0.001***
Epidermal Growth Factor Receptor	71.57 ± 22.21	85.96 ± 15.26	<0.001***
Uric Acid	397.54 ± 134.16	320.96 ± 102.70	0.002**

### The community composition and structure of the microbiome in the two groups

**Fig. 1:**
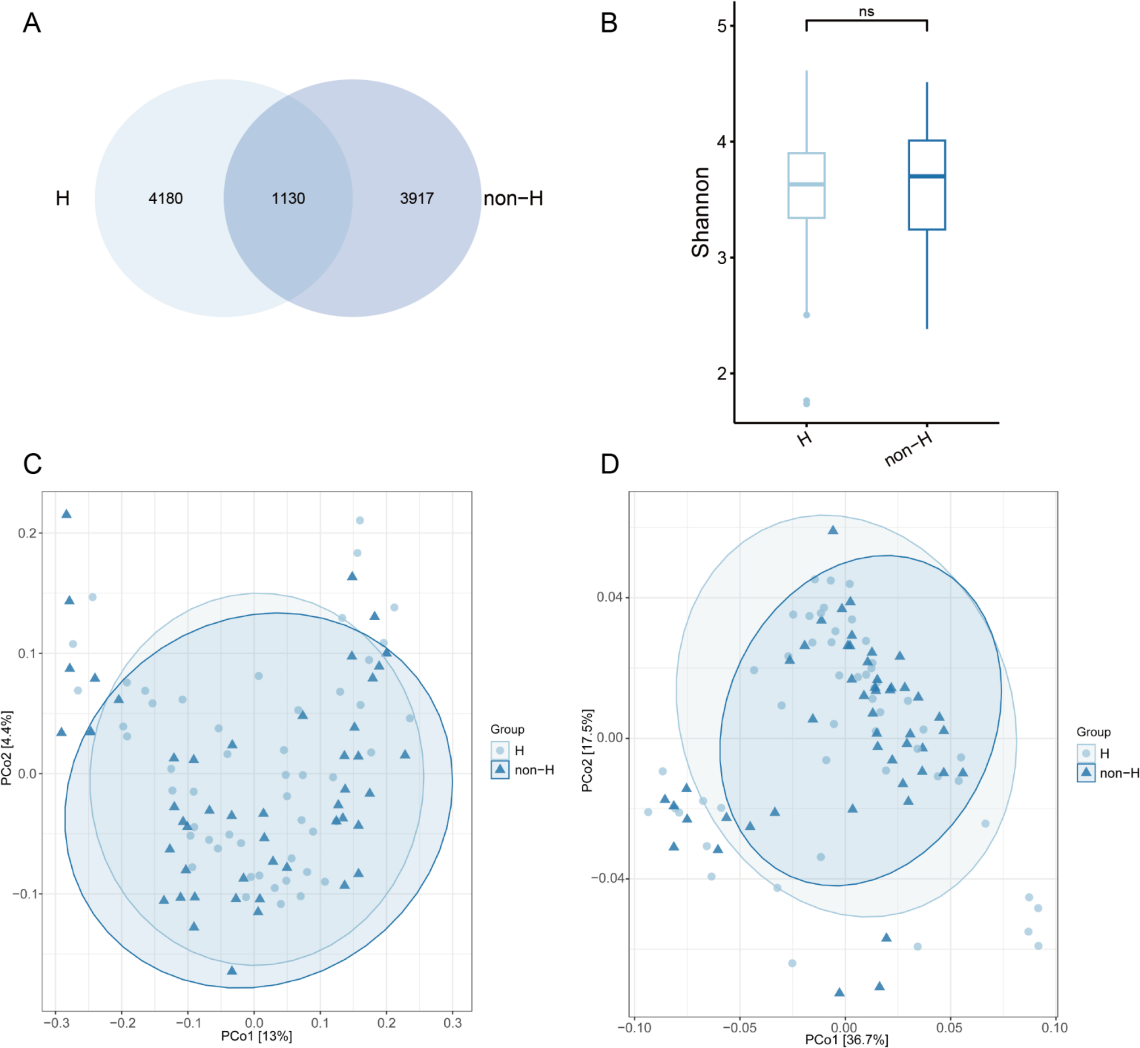
Overview of the microbiota structure. (**A**) Venn plot illustrating the unique and shared ASVs among the two groups. (**B**) Comparations of Shannon Index between the two groups. (**C**) and (**D**) Beta diversity between the two groups using Unweighted Unifrac PCoA and Weighted Unifrac PCoA, respectively

The 16 S rRNA gene sequencing data of two groups of patients were compared and analyzed to illustrate the differences between the two groups. According to the Venn diagram (Fig. 1A), H group and non-H group had 4180 and 3917 unique ASVs, respectively, with 1130 ASVs common to both groups. Through α and β diversity analysis, the richness and composition of the microbial communities in the two groups were revealed. However, as shown in Fig. 1B-D, there was no significant difference in α and β diversity between the two groups.

**Fig. 2:**
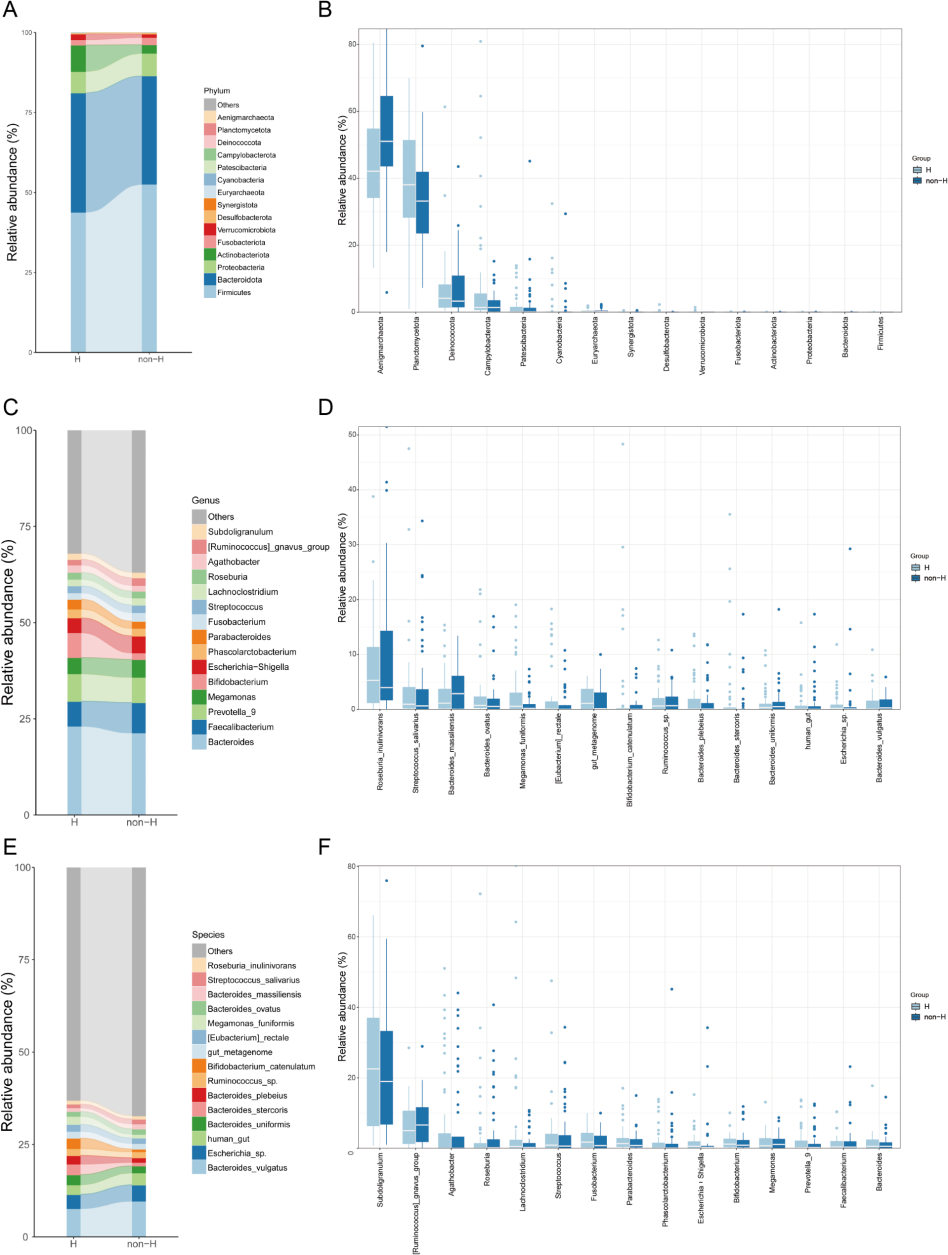
Bar plot and box plot of the top 10 most abundant species at different levels. (**A**) and (**B**) Phylum level. (**C**) and (**D**) Genus level. (**E**) and (**F**) Species level

Figure 2 shows the composition of the colonic microbiota in patients with H-type and non-H-type hypertension at different taxonomic levels. At the phylum level, the four most abundant phyla in both groups were Firmicutes, Bacteroidota, Proteobacteria, and Fusobacteriota (Fig. 2A and B); at the genus level, the abundance of Bifidobacterium was higher in the H group compared to the non-H group (Fig. 2C and D); at the species level, compared to the H group, the non-H group had a higher abundance of Bacteroides vulgatus, a lower abundance of Bacteroides stercoris, and a lower abundance of Bacteroides plebeius (Fig. 2E and F).

### LDA analysis reveals the phylogenetic and taxonomic profiles in microbiome among the two groups

**Fig. 3:**
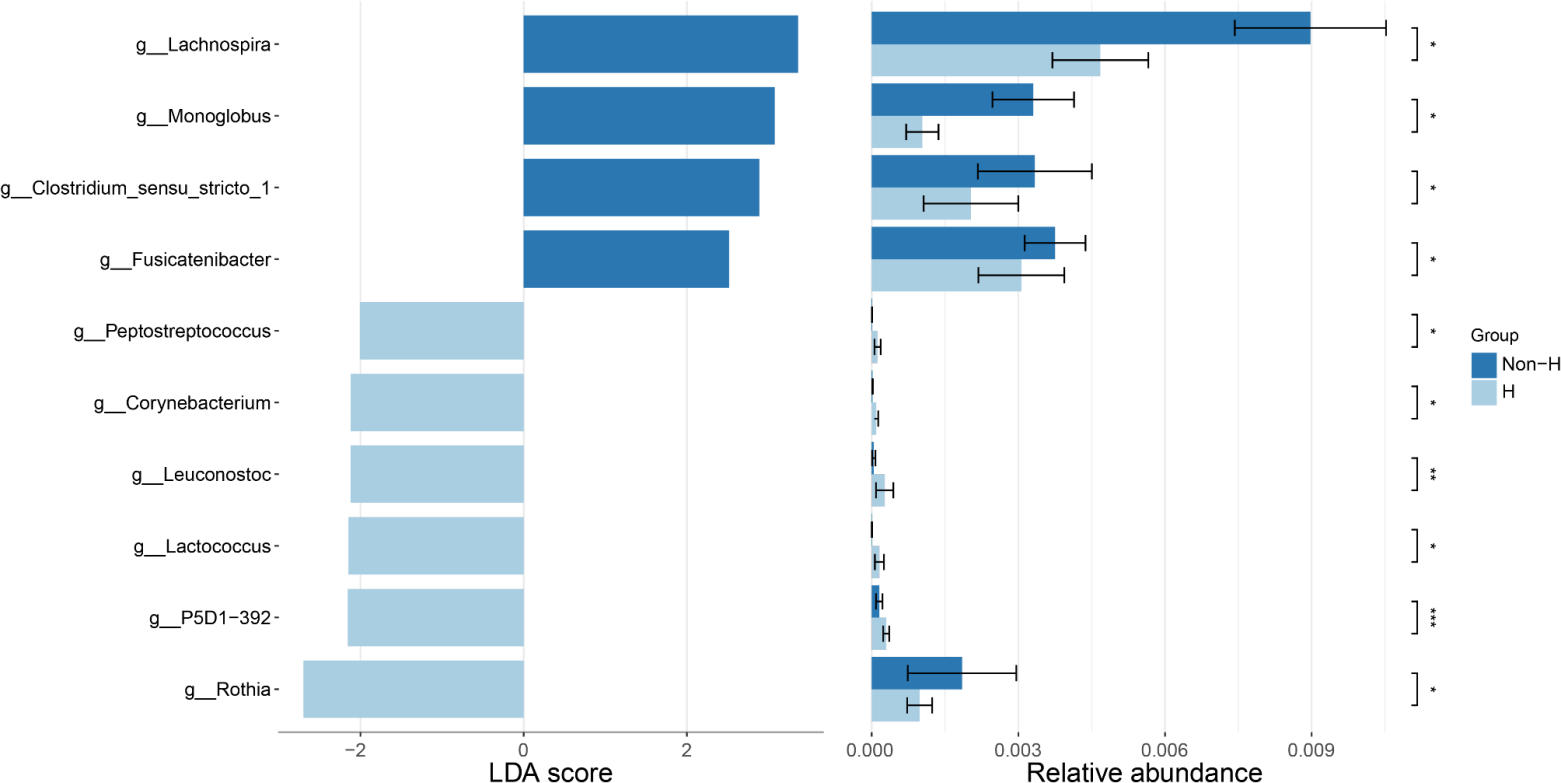
Gut microbiota difference between the two groups were identified with a LEfSe analysis with LDA score threshold > 2.0

Subsequently, we aimed to identify differences in certain taxonomic groups between the H and non-H groups. Therefore, we conducted a LEfSe analysis, utilizing effect size measurements to enrich bacterial taxa with different abundances between the two groups. At a significance threshold (*p* < 0.05) and an LDA score > 2, Fig. 3 shows the differences in abundance at the genus level between the two groups. Compared to the H group, the non-H group has higher abundances of Lachnospira, Monoglobus, Clostridium sensu stricto 1, and Fusicatenibacter.

### Differences in serum and fecal metabolites between the two groups of patients

To further explore the differences in metabolites between the two groups, we tested various short-chain fatty acids, TAMO, and PAGln metabolites in the serum and feces of the two groups of patients. In the serum of both groups, the concentrations of Acetate and Isobutyrate were significantly higher in the H group (*P* < 0.05), while the concentrations of other short-chain fatty acids, TAMO, and PAGln did not show significant differences between the two groups (*P* > 0.05) (Table 3). In the feces of the two groups, the concentration of Malonate was significantly higher in the non-H group (*P* < 0.05) (Table 4).

**Table 3:** Metabolites in the serum of two groups of patients

Feature	H group	Non-H group	*P*
TAMO	345.07 ± 613.08	248.39 ± 276.99	0.65
PAGln	786.27 ± 735.55	666.06 ± 547.62	0.68
Acetate	136.38 ± 49.90	112.49 ± 38.15	0.03*
Propionate	4.51 ± 3.65	4.44 ± 3.59	0.33
Isobutyrate	0.85 ± 0.66	0.65 ± 0.44	0.03*
Butyrate	0.73 ± 1.79	0.55 ± 0.41	0.61
Malonate	0.37 ± 0.34	0.31 ± 0.19	0.40
Succinate	4.54 ± 2.69	4.36 ± 2.48	0.75
2-Methylbutyrate	0.74 ± 3.21	0.25 ± 0.13	0.29
Isovalerate	0.79 ± 0.52	0.72 ± 0.28	0.80
Methylmalonic Acid	0.48 ± 0.95	0.42 ± 0.54	0.65
Glutarate	0.86 ± 0.94	0.79 ± 0.74	0.76
Valerate	0.18 ± 0.57	0.10 ± 0.13	0.17

**Table 4:** Metabolites in the feces of two groups of patients

Feature	H group	Non-H group	*P*
Acetate	1615.80 ± 1026.83	1478.57 ± 1039.81	0.49
Propionate	422.24 ± 273.27	419.84 ± 295.81	0.78
Isobutyrate	37.42 ± 40.53	35.34 ± 35.24	0.91
Butyrate	192.40 ± 182.21	173.54 ± 184.11	0.54
Malonate	0.11 ± 0.15	0.12 ± 0.47	<0.001***
Succinate	28.94 ± 83.38	9.81 ± 25.16	0.88
Methylbutyrate	20.97 ± 25.97	17.94 ± 19.69	0.91
Isovalerate	31.91 ± 39.35	28.09 ± 31.38	0.96
Glutarate	1.80 ± 2.76	1.63 ± 1.87	0.26
Valerate	36.54 ± 42.74	41.45 ± 59.75	0.88
4-Methylvalerate	1.81 ± 6.43	1.38 ± 2.28	0.33
Caproate	6.21 ± 14.62	9.82 ± 34.09	0.75

### The relationship between gut microbiota and metabolites in serum and feces in the two groups of patients

**Fig. 4:**
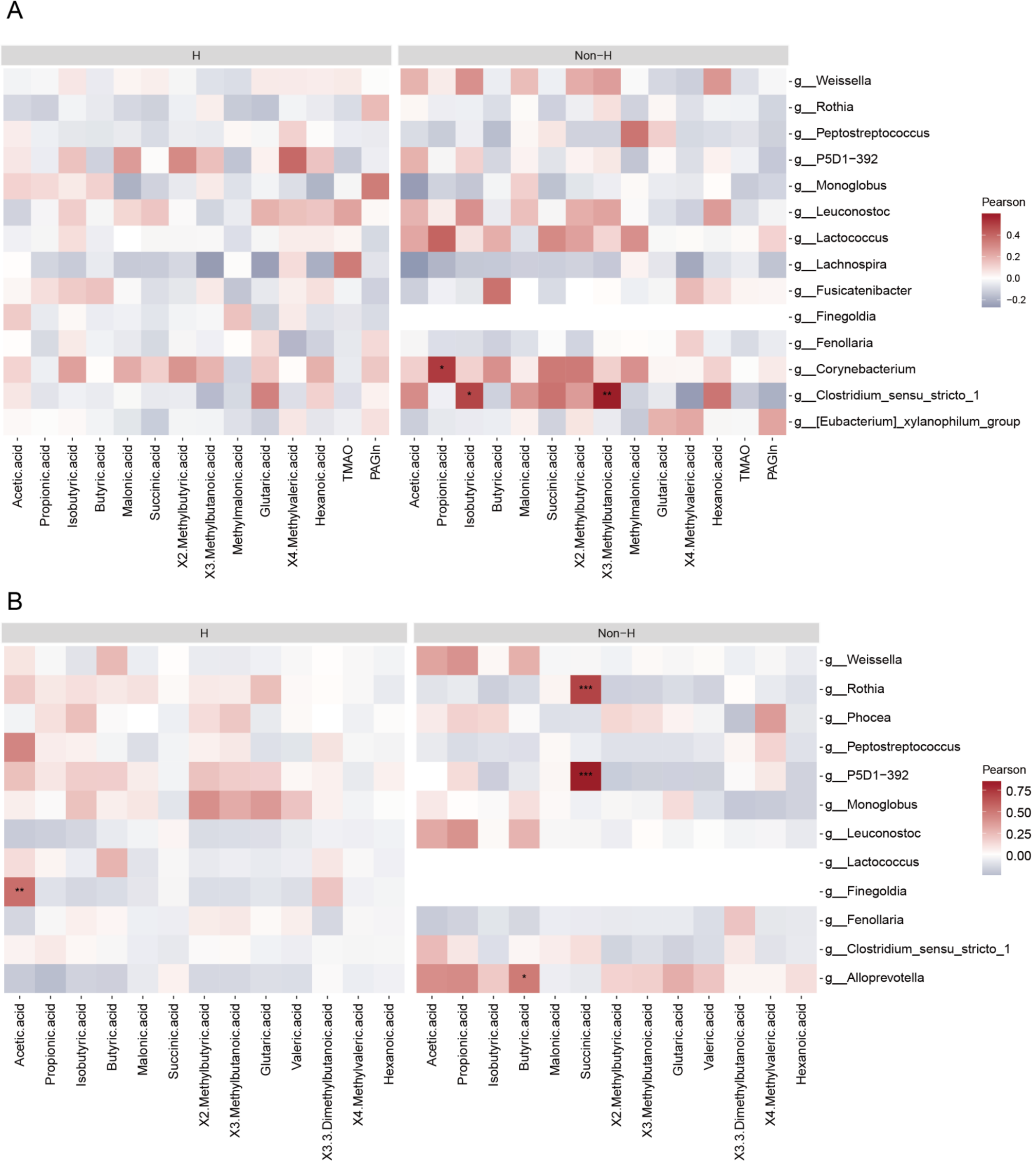
The correlations between metabolites in feces (**A**) and serum (**B**) and differentially abundant ASVs identified by LEfSe analysis

To further explore the relationship between gut microbiota and metabolites in serum and feces in the two groups, we analyzed the correlation between differentially abundant species from the LEfSe analysis and metabolites. As shown in Fig. 4A, in the H group, Finegoldia is positively correlated with fecal acetic acid. In the Non-H group, Rothia and P5D1-392 are positively correlated with fecal succinic acid, and Alloprevotella is positively correlated with fecal butyric acid. Figure 4B shows the relationship between gut microbiota and serum metabolites. In the Non-H group, Corynebacterium is positively correlated with propionic acid, and Clostridium sensu stricto 1 is positively correlated with both isobutyric acid and 3-methylbutanoic acid.

### The co-occurrence network of gut microbiota in the two groups of patients

**Fig. 5:**
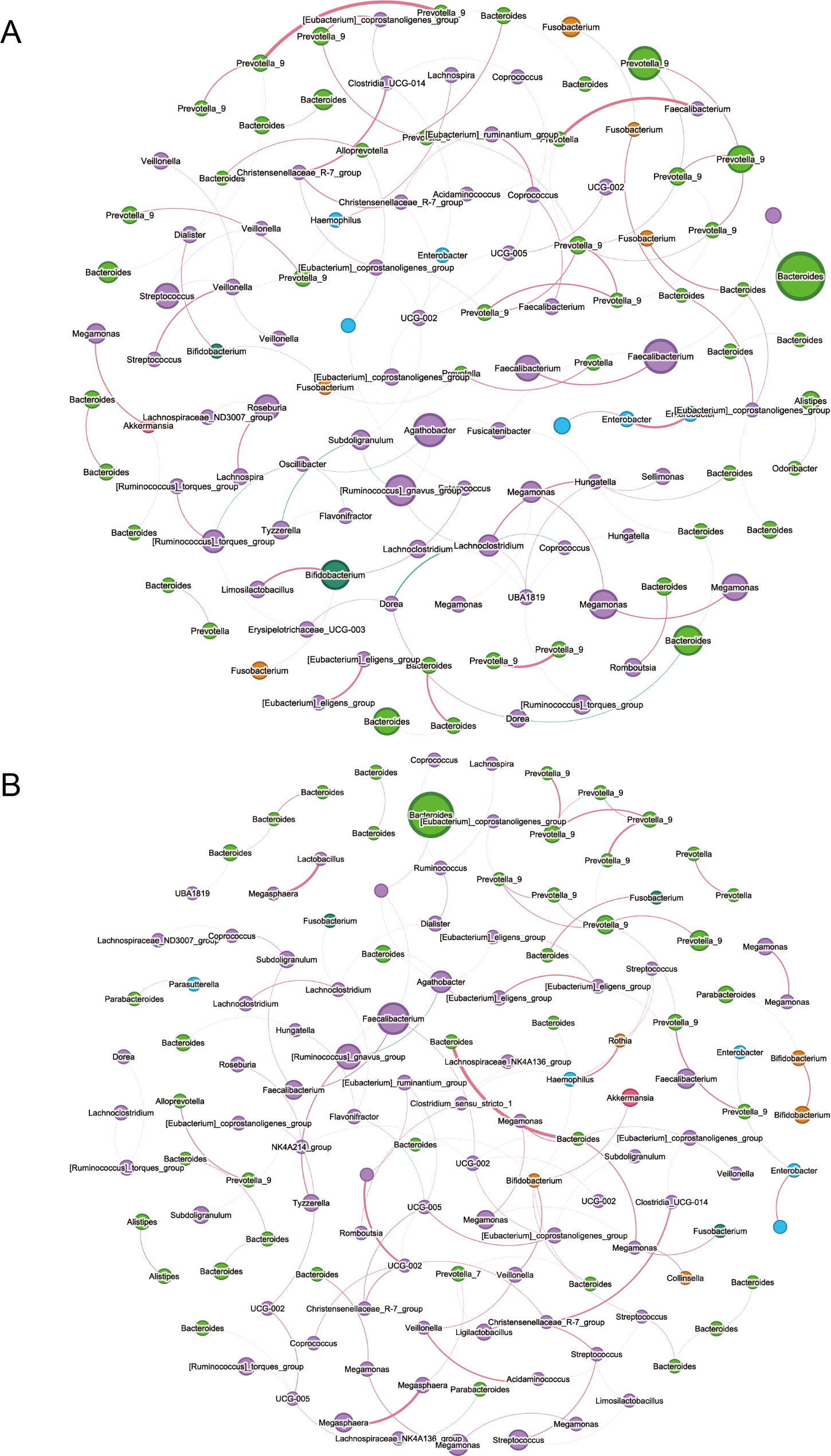
Co-occurrence network analysis for the two groups. (**A**) H group. (**B**) Non-H group. Node size was presented by its degree. The node colors were based on phylum level

To further explore key taxa between the two groups of patients, we constructed a microbial co-occurrence network using Spearman correlations among different taxa (Fig. 5). Compared to the H group, the interactions among gut microbiota in the Non-H group are more complex. Certain species act as key nodes in the network. For example, in the H group, Hungatella and Enterobacter serve as important nodes connecting with Bacteroides (Fig. 5A). In the Non-H group, Faecalibacterium and Agathobacter are crucial nodes connecting with Ligilactobacillus (Fig. 5B).

### Differences in microbial metabolic pathways between the two groups of patients

**Fig. 6:**
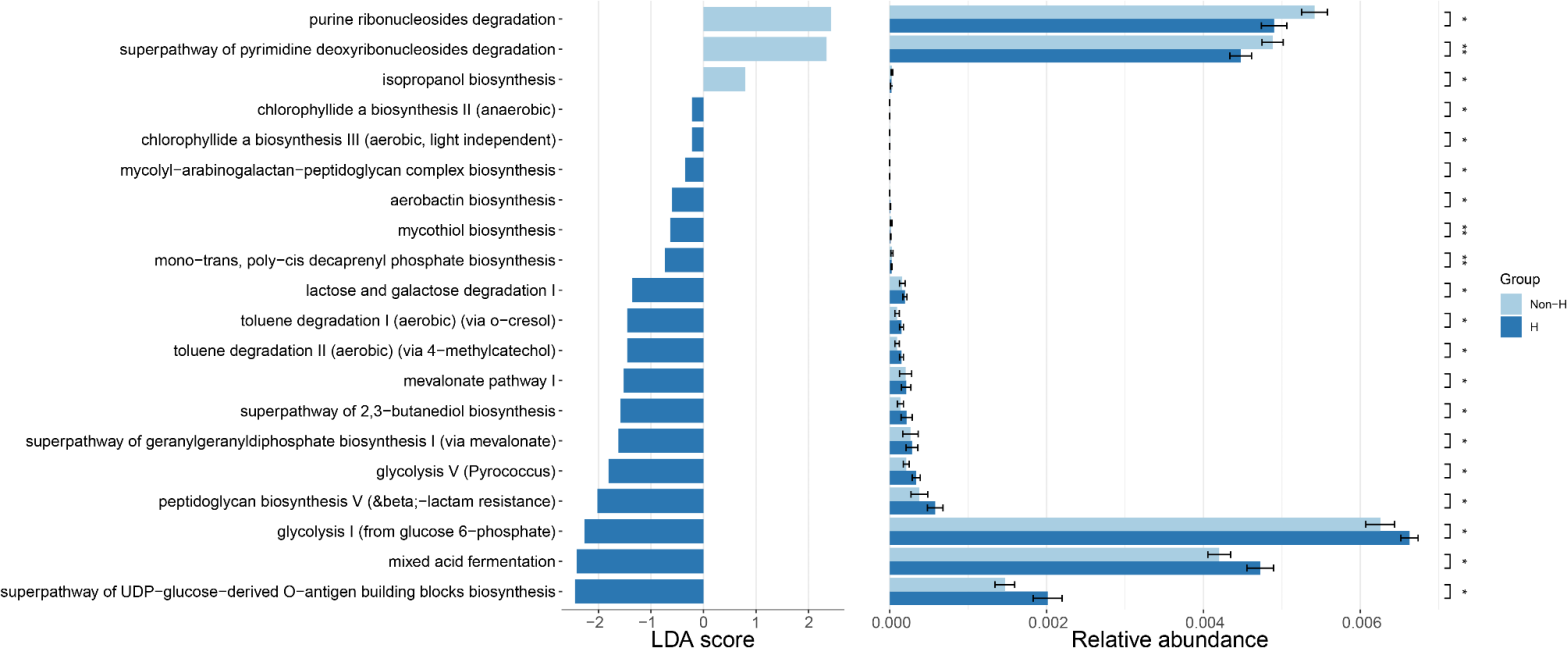
The results of KEGG annotations by PICRUSt2-predicted based on 16 S rRNA gene sequencing data of the two groups

PICRUSt 2 was used for predicting functional abundances, and the Kyoto Encyclopedia of Genes and Genomes (KEGG) database was subsequently used for functional pathway annotation. As shown in Fig. 6, we compared the differences in functional pathways between the two groups at KEGG. The Non-H group exhibited higher levels of biological processes such as purine ribonucleosides degradation, superpathway of pyrimidine deoxyribonucleosides degradation, and isopropanol biosynthesis compared to the H group. In contrast, peptidoglycan biosynthesis V, glycolysis I, mixed acid fermentation, and superpathway of UDP-glucose-derived O-antigen building blocks biosynthesis were higher in the H-type hypertension group than in the Non-H group.

## Discussion

Analysis of data from two groups of patients reveals that the proportion of H-type hypertensive patients with a history of smoking and alcohol abuse is higher. Studies have shown that the risk of gene mutations in smokers is significantly higher than in non-smokers [17]. Additionally, interaction analysis suggests that the ALDH2 rs190914158 mutation, in interaction with smoking, may contribute to the development of H-type hypertension. It is reported that smoking can damage the heart and vascular system, increasing the risk of arteriosclerosis and arterial wall thickening, leading to hypertension. Previous research indicates that the risk of hypertension in smokers is 30–50% higher than in non-smokers [17]. Epidemiological surveys indicate that the smoking rate is higher among H-type hypertensive patients, and the progression of H-type hypertension is faster compared to non-smokers. According to data from the American Heart Association, the smoking rate among family members of H-type hypertensive patients is about 27%, compared to only 16% among non-H-type hypertensive family members [18].

Research has found that hypertension patients exhibit gut dysbiosis, characterized by a decrease in gut microbiota diversity and abundance, an increase in the Firmicutes/Bacteroidetes ratio, a reduction in bacteria producing acetate and butyrate, and an increase in lactate-producing bacteria [19–21]. This has also been validated in animal models [22, 23]. Sequencing results of the two groups’ microbiota show no significant differences in α and β diversity, but at the phylum level, non-H group patients have higher Firmicutes abundance and lower Actinobacteriota abundance. At the genus level, the H-type group has a higher abundance of Bifidobacterium compared to the non-H-type group. At the species level, the non-H group has a higher abundance of Bacteroides_vulgatus, and lower abundances of Bacteroides_stercoris and Bacteroides_plebeius. In the serum of both groups, acetate and isobutyrate concentrations are significantly higher in the H group (*P* < 0.05), and in the feces, malonate concentration is significantly higher in the non-H group. Studies have found that patients with hypertension have higher abundances of Aminobacterium, Cutibacterium, and Muribaculaceae, while Ruminococcus and Fastidiosipila are less abundant. The concentrations of acetate and butyrate are increased in the plasma of hypertensive patients [24]. Gut microbiota dysbiosis promotes hypertension and is associated with a decrease in bacteria that produce short-chain fatty acids [25].

Rothia is involved in the metabolism of organic acids within the intestine, including the production and utilization of succinate. Through metabolic pathways, Rothia can convert certain substrates into succinate, thereby influencing the acid-base balance and microecological environment of the intestine [26]. Alloprevotella is a genus of gut bacteria belonging to the family Prevotellaceae. Alloprevotella may be involved in the production of short-chain fatty acids, including butyrate. It promotes the generation of butyrate by metabolizing substrates such as dietary fibers, thereby contributing to the maintenance of gut health [27].

Research has found that the co-occurrence network of fecal microbiota in non-H-type hypertension is more complex, which to some extent indicates that the level of Hcy may affect the gut microbiota. When serum Hcy levels are lower, the contribution network of fecal microbiota is more intricate, which may be related to several factors. Lower Hcy levels typically reflect a good nutritional status, especially with adequate supplies of nutrients like vitamins B6, B12, and folic acid, which help promote the growth of diverse microorganisms in the gut [28, 29]. Low Hcy levels may also be associated with a reduced inflammatory state, leading to less dysbiosis in the gut microbiota and enhancing its diversity and complexity. Moreover, lower levels of Hcy may facilitate more frequent interactions between microorganisms, resulting in a more complex ecosystem. Therefore, maintaining appropriate Hcy levels is important for promoting gut health and the diversity and functionality of the microbiota [30].

In patients with H-type hypertension, there is a significant association between elevated levels of acetate and isobutyrate in serum and changes in the microbiome. Studies indicate that specific gut microbiota, such as a reduction in certain Bacteroides species, may lead to an imbalance in the metabolism of short-chain fatty acids (SCFAs), thereby affecting the host’s metabolic state [31, 32]. The increase in acetate and isobutyrate may exert effects through several mechanisms, such as enhancing endothelial function, regulating inflammatory responses, and improving gut barrier integrity, thus reducing the risk of hypertension [33]. Existing research supports the anti-inflammatory properties of SCFAs and their ability to promote nitric oxide production, which plays a significant role in the pathophysiology of hypertension [34]. Therefore, the microbiome and its metabolites may have a crucial role in the onset and progression of H-type hypertension, and further research will help elucidate these mechanisms and provide new insights for the prevention and treatment of hypertension.

The results of this study found that the level of purine ribonucleotide degradation pathways was elevated in the non-H type hypertension group. Currently, the relationship between purine ribonucleotide degradation and hypertension has attracted researchers’ attention. Adenosine, as a product of purine metabolism, can promote vasodilation by acting on specific receptors (such as A1 and A2 receptors), thereby lowering blood pressure [35, 36]. In addition, adenosine may also affect renal function, regulating body water and electrolyte balance, which is crucial for blood pressure control. Moreover, intermediates of purine metabolism may be involved in regulating inflammatory responses, which are closely related to hypertension [37]. Furthermore, the degradation of purine nucleotides may also be associated with oxidative stress levels, which is considered one of the important mechanisms of hypertension [37]. Although existing studies have indicated a certain association between the two, the specific mechanisms still need to be explored in depth to better understand the role of purine metabolism in the management of hypertension. Therefore, to some extent, we can differentiate between H type and non-H type hypertension by measuring the levels of purine ribonucleotide degradation pathways in hypertensive patients, which may provide certain assistance in the diagnosis and treatment of patients.

## Conclusions and limitations

Microbial sequencing and metabolites testing revealed distinct differences between the H-type and non-H hypertensive groups, with unique gut microbiota compositions and metabolite profiles. In addition to the elevated serum acetate in the H group and higher fecal malonate in the non-H group, correlation analysis showed that specific gut microbiota species, such as Finegoldia in the H group and Rothia and Alloprevotella in the non-H group, are associated with different metabolites. These findings underscore the complex relationship between gut microbiota and metabolites in hypertension.

Despite these findings, the study has several limitations. First, the sample size of 100 hypertensive patients may limit the representativeness and generalizability of the results. Additionally, although key factors such as gender, age, and medical history were controlled for, other unmeasured variables (e.g., diet, genetics) could still impact microbiota and metabolite differences. The cross-sectional design of the study also prevents establishing causal relationships between gut microbiota and hypertension. Furthermore, the study focused on a limited set of fatty acids and metabolites. Future research should include more comprehensive metabolomic analyses and standardized methodologies to better understand the gut microbiota interactions in H-type hypertension patients. Acknowledging these limitations will help refine future study designs.
